# Measurement of epicardial adipose tissue using non-contrast routine chest-CT: a consideration of threshold adjustment for fatty attenuation

**DOI:** 10.1186/s12880-022-00840-3

**Published:** 2022-06-25

**Authors:** Lekang Yin, Cheng Yan, Chun Yang, Hao Dong, Shijie Xu, Chenwei Li, Mengsu Zeng

**Affiliations:** 1 Department of Radiology, Zhongshan Hospital, Fudan University, No. 180 Fenglin Rd, Xuhui District, Shanghai, 200032 China; 2 Department of Radiology, First People’s Hospital of Xiaoshan District, Hangzhou, 311200 China; 3 Shanghai United Imaging Healthcare Co., Ltd, No. 2258 Chengbei Rd., Jiading District, Shanghai, 201807 China; 4 Shanghai Institute of Medical Imaging, 180 Fenglin Road, Xuhui District, Shanghai, China; 5 Department of Medical Imaging, Shanghai Medical College, Fudan University, No. 138 Yi xue yuan Road, Shanghai, 200032 China

**Keywords:** Adipose tissue, Computedtomography angiography, Coronary artery disease, Pericardium, Multidetector computed tomography

## Abstract

**Background:**

Epicardial adipose tissue (EAT) is known as an important imaging indicator for cardiovascular risk stratification. The present study aimed to determine whether the EAT volume (EV) and mean EAT attenuation (mEA) measured by non-contrast routine chest CT (RCCT) could be more consistent with those measured by coronary CT angiography (CCTA) by adjusting the threshold of fatty attenuation.

**Methods:**

In total, 83 subjects who simultaneously underwent CCTA and RCCT were enrolled. EV and mEA were quantified by CCTA using a threshold of (N30) (− 190 HU, − 30 HU) as a reference and measured by RCCT using thresholds of N30, N40 (− 190 HU, − 40 HU), and N45 (− 190 HU, − 45 HU). The correlation and agreement of EAT metrics between the two imaging modalities and differences between patients with coronary plaques (plaque ( +)) and without plaques (plaque ( −)) were analyzed.

**Results:**

EV obtained from RCCT showed very strong correlation with the reference (r = 0.974, 0.976, 0.972 (N30, N40, N45), *P* < 0.001), whereas mEA showed a moderate correlation (r = 0.516, 0.500, 0.477 (N30, N40, N45), *P* < 0.001). Threshold adjustment was able to reduce the bias of EV, while increase the bias of mEA. Data obtained by CCTA and RCCT both demonstrated a significantly larger EV in the plaque ( +) group than in the plaque ( −) group (*P* < 0.05). A significant difference in mEA was shown only by RCCT using a threshold of N30 (plaque ( +) vs ( −): − 80.0 ± 4.4 HU vs − 78.0 ± 4.0 HU, *P* = 0.030). The mEA measured on RCCT using threshold of N40 and N45 showed no significant statistical difference between the two groups (*P* = 0.092 and 0.075), which was consistent with the result obtained on CCTA (*P* = 0.204).

**Conclusion:**

Applying more negative threshold, the consistency of EV measurements between the two techniques improves and a consistent result can be obtained when comparing EF measurements between groups, although the bias of mEA increases. Threshold adjustment is necessary when measuring EF with non-contrast RCCT.

## Background

The visceral fat located between the myocardial surface and the visceral layer of the pericardium, known as epicardial adipose tissue (EAT), is well known as an important imaging indicator for cardiovascular risk stratification [1]. Evidence from the last two decades has shown that EAT plays various regulatory roles relating to cardiac biology, including atherosclerosis progression, atrial fibrillation and heart failure [2–5]. EAT also acts as a paracrine or vasocrine organ by locally releasing bioactive cytokines into the adjacent interstitium of the myocardium and coronary arteries [2–5]. The underlying complex and important functions related to metabolic, thermogenic and mechanical properties and the relationship to noncardiac organs and systemic diseases are receiving increasing attention [6]. The amount of EAT has been reported to be a good predictor of the risk of metabolic syndrome and an appealing biomarker to evaluate the efficacy of certain therapies, such as pharmacological therapies for obesity, dyslipidemia and type 2 diabetes mellitus [6–8]. On the other hand, cardiac complications have been demonstrated in the coronavirus disease 2019 (COVID-19) pandemic [9]. EAT has a high level of angiotensin-converting enzyme 2 (ACE2) expression, so it is suspected to be an important mediator of the inflammatory response in the myocardium after SARS-CoV-2 infection [10, 11]. Thus, an approach to quantify EAT consistently and economically would be advantageous.

Cardiac examinations such as echocardiography, coronary CT angiography (CCTA) and the coronary calcium score (CCS) are currently the most common imaging techniques used to assess the amount of EAT [12, 13]. However, these cardiac examinations have relatively strict clinical indications, limiting the amount of data that can be used to assess EAT. Actually, routine chest CT (RCCT) imaging can also be used to quantify the volume of EAT (EV), especially the routinely performed non-contrast RCCT. When using the same threshold for fatty attenuation, the EV measured by RCCT correlates well with that measured by CCTA, but it is overestimated [14]. In addition, the use of EAT attenuation (EA) as a measure of fat composition [15, 16] has not been compared between the two imaging modalities. Therefore, the present study aimed to determine whether EV and mean EA (mEA) measured with RCCT by adjusting the threshold of fatty attenuation could be more consistent with those measured with CCTA on the same scanner.

## Materials and methods

### Subject selection

All subjects who underwent coronary CT angiography (CCTA) and non-contrast RCCT simultaneously for health check-ups in our center from January 2019 to August 2020 were retrospectively investigated. Those who underwent surgery or invasive procedures of the lung, mediastinum and heart were excluded. A total of 83 subjects were ultimately enrolled in this study. Information about sex, age, diabetes, dyslipidemia, and hypertension was collected from their records. This retrospective study was approved by the institutional review board. All participants were fully informed and agreed that their medical records would be anonymized for research purposes.

### CT examination procedure

CCTA and RCCT scanning were performed on the same 320-slice CT scanner (uCT960 + , Shanghai United Imaging Healthcare) sequentially without changing the position. The non-ECG-gated RCCT parameters were as follows: 120 kVp; 300 mAs; detector collimation, 160 × 0.5; pitch, 1.0938; rotation time, 0.5 s; matrix size, 1024 × 1024; field of view, 350 mm; and slice thickness, 1.0 mm, covering the scanning range from the lung apices to the bases. Subsequently, CCTA was performed using a breath-hold prospective axial ECG-triggered acquisition protocol. For patients with heart rate > 65 beats/min, metoprolol was taken orally approximately 1–1.5 h before CCTA examination. Sublingual nitroglycerine (0.5 mg) was administered 5 min before scanning, except in the case of contraindications. Intravenous injection of iodinated contrast medium was injected through the right cubital vein with a double cylinder high pressure syringe (370 mgI/ml, flow rate: 4.0–5.0 ml/s, the total amount of injection was 0.8 ml/kg) followed by saline (25 ml) injection at the same flow rate. The scan was obtained from the carina to the bottom of the heart, and Bolus Tracking automatic trigger scanning technology was used. The monitoring layer was located at the center of the scanning range, the ROI was placed at the center of the descending thoracic aorta, the triggering threshold was set at 120 HU, and the scanner was delayed 6 s to start scanning automatically after reaching the threshold. The parameters were as follows: 100 kVp; 120 mAs; detector collimation, 320 × 0.5; pitch, 1.0938; rotation time, 0.25 s; matrix size, 512 × 512; field of view, 350 mm; and slice thickness, 0.5 mm.

### Assessment of CCTA

All the images were imported from the Picture Archiving and Communication System to the postprocessing workstation (uWS-CT, R004, Shanghai United Imaging Health care). Both the cross-sections and longitudinal reconstructed images were visually inspected to detect coronary plaques by two radiologists with 10 and 15 years of experience in cardiac imaging analysis. The presence of atherosclerotic plaques and stenosis grading were evaluated based on the 18-segment model recommended by Society of Cardiovascular Computed Tomography [17]. Stenosis grading used the 6-level scale as follows: 1-Normal, the absence of plaques and no luminal stenosis; 2-Minimal, plaques with < 25% stenosis; 3-Mild, 25% to 49% stenosis; 4-Moderate, 50% to 69% stenosis; 5-Severe, 70% to 99% stenosis; 6-Occluded. Structures clearly assignable to the vessel wall on at least two views with densities less than the lumen contrast were classified as noncalcified plaques. Any structure with a density ≥ 130 HU that could be visualized separately from the contrast-enhanced coronary lumen was defined as a calcified plaque, which included calcified and partially calcified plaques. Patients with any form of plaque, including noncalcified and calcified plaques, were defined as the plaque-positive (plaque ( +)) group. The other patients were defined as the plaque-negative (plaque ( −)) group.

### Measurement of EAT

**Fig. 1:**
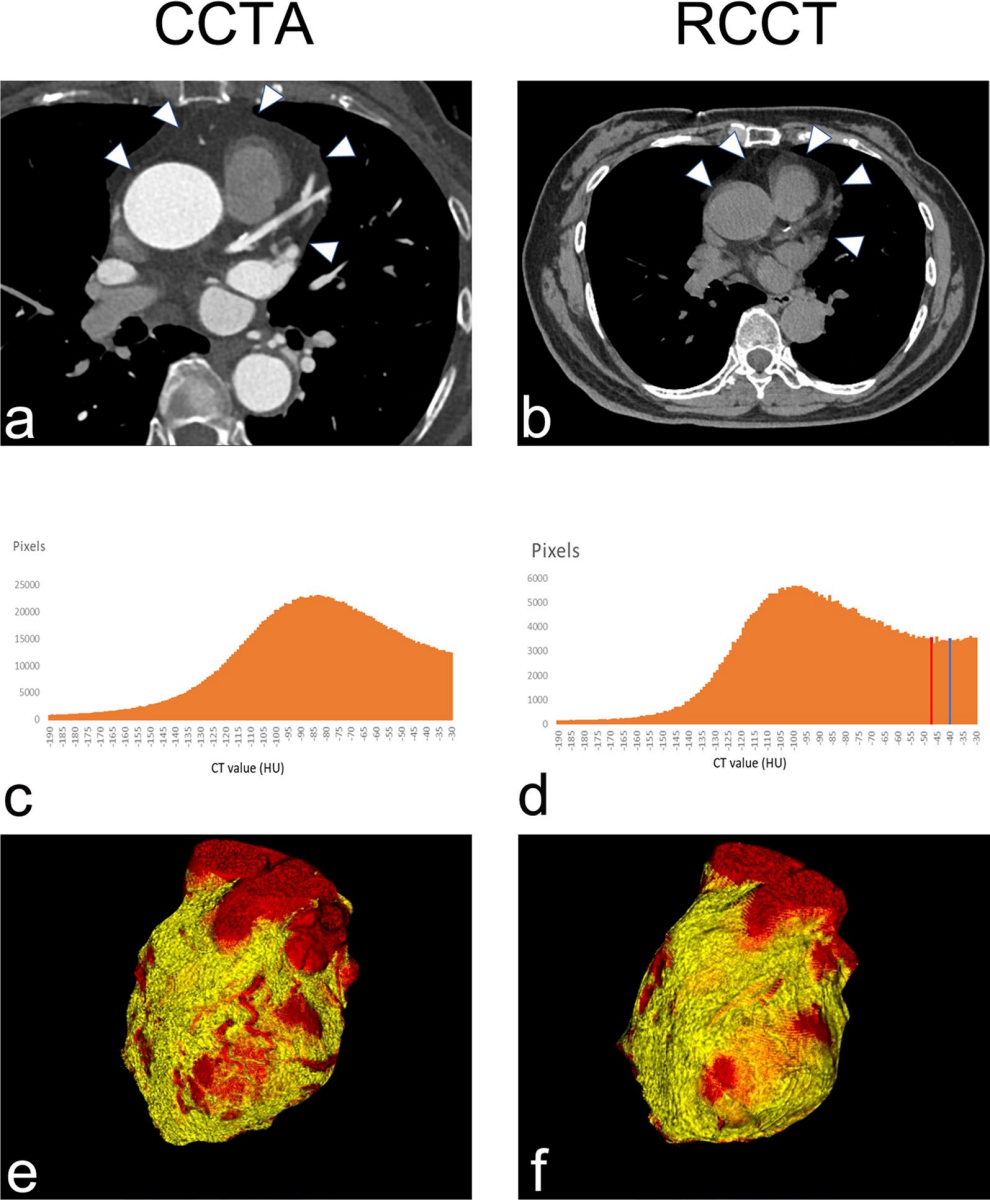
Visualization of the EAT in axial images, histograms and volume rendering. The pericardial structure (white arrowhead) in RCCT could be identified as clearly as that in CCTA (**a**, **b**). The similar pattern of CT value histograms extracted from pericardium segmentation from CCTA and RCCT both indicate that the adjustment of upper thresholds had a more obvious influence on the precision of fat volume measurements (**c**, **d**). The blue and red lines indicate the CT attenuation of − 40 HU (N40) and − 45 HU (N45), respectively. Volume rendering of the EAT and heart are displayed using the same threshold of (− 190 HU, − 30 HU) (**e**, **f**)

EAT was defined as the visceral fat between the myocardial surface and the visceral layer of the pericardium. (Fig. 1a, b). The pericardium was manually traced from the bifurcation of the pulmonary trunk to the end of the pericardial sac. The volume of the whole heart and the frequency table of CT values within the heart were generated and exported to a personal computer. The threshold of fat tissue was applied to define the fat-containing voxels. The EV (reported in cm^3^) equals the product of the volume of a single voxel and the number of fat-containing voxels. The mEA (reported in HU) was defined as the mean attenuation of all fat-containing voxels. An attenuation histogram was reviewed to show the distribution of fat-containing voxels. A threshold of − 190 to − 30 Hounsfield units (HU) (− 190 HU, − 30 HU) was applied to extract the fat-containing voxels for CCTA imaging. For the RCCT, the lower threshold was fixed at − 190 HU, and the upper threshold was adjusted and set at − 30 HU (N30), − 40 HU (N40) and − 45 HU (N45). The measured EV and mEA at the corresponding thresholds were recorded as EV_N30_ and EA_N30_, EV_N40_ and mEA_N40_, EV_N45_ and mEA_N45_, respectively.

### Statistical analysis

Statistical analyses were performed using SPSS version 19.0 for Windows (SPSS Inc., Chicago, IL, USA). The Kolmogorov–Smirnov test was used to check whether the data conformed to normal distribution. Continuous variables with normal distribution are expressed as the mean ± standard deviation (SD), and categorical variables are expressed as N (%). The measurement data from CCTA were compared with those from RCCT using the paired t test. Correlations and agreement of EAT measurements between RCCT and CCTA scans were evaluated using Pearson’s correlation test and Bland–Altman analysis. The difference in EAT according to the presence or absence of coronary atherosclerotic plaques was analyzed using Student’s t-test, Mann–Whitney U test, and the dichotomic variables were analyzed using chi-squared test. A *P* value < 0.05 was considered statistically significant.

## Results

Among all 83 subjects enrolled, 49 subjects (59.0%) had no plaques or luminal stenosis detected by CCTA. Plaques were detected in the other 34 (41.0%) subjects, in which 9 (26.5%) had minimal stenosis, 18 (52.9%) had moderate stenosis, 5 (14.7%) had severe stenosis, and 1 (2.9%) was occluded. Among the patients with plaques, 24 subjects (70.6%) had calcified plaques, and 10 subjects (29.4%) had noncalcified plaques. Regarding the number of involved coronary artery segments, 17 subjects (50.0%) had only 1 segment, 7 subjects (20.6%) had 2 segments, 5 subjects (14.7%) had 3 segments, and 5 subjects (14.7%) had 4 segments. The general characteristics are shown in Table 1.

**Table 1:** Patient characteristics and results of EAT measurements

	Total	Plaque ( +)	Plaque ( −)	*P* value
N	83	34	49	
Age	55.3 ± 7.6	57.0 ± 9.0	54.2 ± 6.4	0.097
BMI	24.5 ± 2.9	24.9 ± 2.9	24.1 ± 2.8	0.221
Gender (Male)	46(55.4)	22(64.7)	24(49.0)	0.156
Hypertension (Yes)	41(49.4)	24(70.6)	17(34.7)	0.001*
Diabetes mellitus (Yes)	42(50.6)	20(58.8)	22(44.9)	0.212
Dyslipidemia (Yes)	37(44.6)	19(55.9)	18(36.7)	0.084
Calcified plaque	\	24(70.6)	\	
Non-calcified plaque	\	10(29.4)	\	
EV (cm^3^)	99.8 ± 37.9	115.6 ± 44.1	88.9 ± 28.6	0.003*
EV_N30_ (cm^3^)	115.0 ± 41.8	131.2 ± 49.6	103.7 ± 31.2	0.008*
EV_N40_ (cm^3^)	103.1 ± 39.9	118.8 ± 47.6	92.2 ± 29.4	0.006*
EV_N45_ (cm^3^)	98.0 ± 38.9	112.9 ± 46.6	87.6 ± 28.6	0.009*
mEA (HU)	− 78.8 ± 4.1	− 79.5 ± 3.8	− 78.3 ± 4.2	0.204
mEA_N30_ (HU)	− 78.8 ± 4.3	− 80.0 ± 4.4	− 78.0 ± 4.0	0.030*
mEA_N40_ (HU)	− 84.0 ± 3.6	− 84.8 ± 3.9	− 83.4 ± 3.3	0.092
mEA_N45_ (HU)	− 86.2 ± 3.3	− 87.0 ± 3.5	− 85.7 ± 3.0	0.075

Data are shown as mean ± SD or number (%)

*EAT* Epicardial adipose tissue; *mEA* mean EAT attenuation; *EV* EAT volume; Threshold of N30 (− 190HU, − 30HU), N40: (− 190HU, − 40HU); N45: (− 190HU, − 45HU)

^*^P value < 0.05 was considered statistically significant

### Comparison of EAT measurements between CCTA and RCCT

**Fig. 2:**
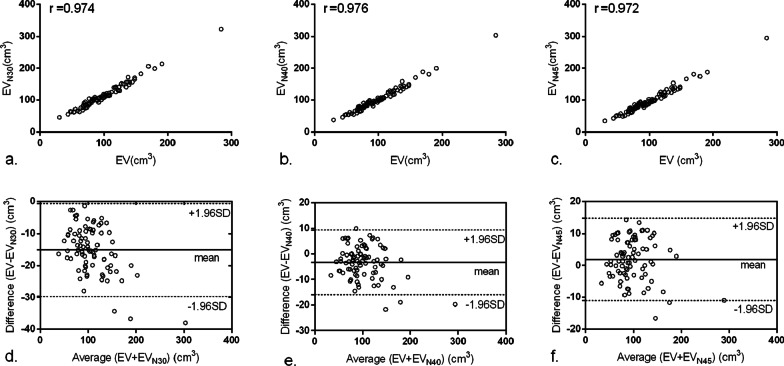
Correlation (**a**–**c**) and Bland–Altman plot (**d**–**f**) for EV between CCTA and RCCT using three thresholds. Mean EV [cm^3^] is plotted against the relative difference of both measurements. Both dotted lines represent the 95% confidence intervals. Threshold of fatty attenuation: N30 (− 190 HU, − 30 HU), N40 (− 190 HU, − 40 HU), and N40 (− 190 HU, − 45 HU)

**Fig. 3:**
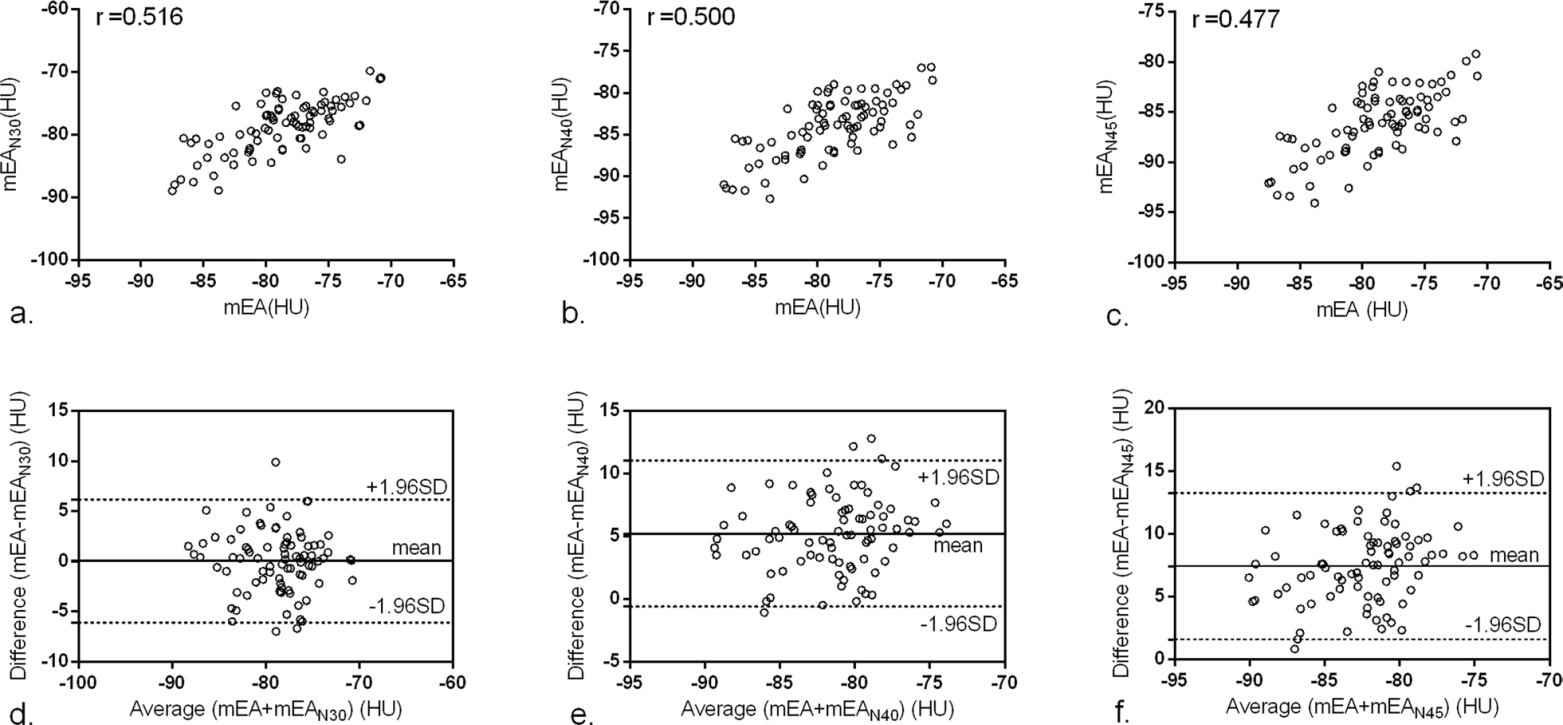
Correlation (**a**–**c**) and Bland–Altman plot (**d**–**f**) for EA between CCTA and RCCT using three thresholds. Mean EA [HU] is plotted against the relative difference of both measurements. Both dotted lines represent the 95% confidence intervals. Threshold of fatty attenuation: N30 (− 190 HU, − 30 HU), N40 (− 190 HU, − 40 HU), and N40 (− 190 HU, − 45 HU)

Visually, the pericardial structure in RCCT could be identified as clearly as in CCTA (Fig. 1a, b) and the EAT displayed in volume rendering of RCCT was obviously more than that of CCTA (Fig. 1e, f.). An attenuation histogram of fat tissue revealed similar curve patterns showing the frequency with higher attenuation was approximately 20–30 times higher than that of the lower attenuation (Fig. 1c, d). When using the same threshold of (− 190 HU, − 30 HU), EV measurements showed strong correlation (r = 0.974), while the correlation of mEA was moderate (r = 0.516) (Figs. 2a, 3a). Bland–Altman analysis showed that the mean difference (95% LoA) of EV and EV_N30_ was − 15.2 (− 29.7 to − 0.6) cm^3^, suggesting an overestimate of approximately 15% in RCCT compared with CCTA using the same threshold (Fig. 2c). While the agreement of mEA between CCTA and RCCT was good, the mean difference (95% LoA) of mEA and mEA_N30_ was 0 (− 6.1 to 6.2) HU (Fig. 3c).

### Effect of threshold adjustment on EAT measurement

After adjustment, EV_N40_ and EV_N45_ still correlated strongly with the EV in CCTA (r = 0.976, 0.972, *P* < 0.001) (Fig. 2b, c). Bland–Altman analysis showed that the mean differences (95% LoA) of EV and EV_N40_ and EV_N45_ were − 3.3 (− 15.9 to 9.4) cm^3^ and 1.9 (− 11.1 to 14.8) cm^3^, respectively (Fig. 2d, f.). Adjusting the threshold reduced the bias of FFV from − 15.2 cm^3^ to − 3.3 cm^3^ or 1.9 cm^3^. The mEA in RCCT using adjusted thresholds (mEA_N30_, mEA_N40_, and mEA_N45_) correlated moderately with the EV in CCTA (r = 0.516, 0.500, 0.477, *P* < 0.001) (Fig. 3a–c). The mean differences (95% LoAs) of mEA and mEA_N30_, mEA_N40_, and mEA_N45_ were 0 (− 6.1 to 6.2) HU, 5.2 (− 0.6 to 11.1) HU, and 7.4 (1.6 to 13.3) HU, respectively (Fig. 3d–f.). Although there was moderate correlation and agreement between the mEA measured with the two imaging modalities, the correlation coefficient decreased, and the bias increased accordingly after threshold adjustment.

### Comparison of EAT measurements based on the presence of plaques

**Fig. 4:**
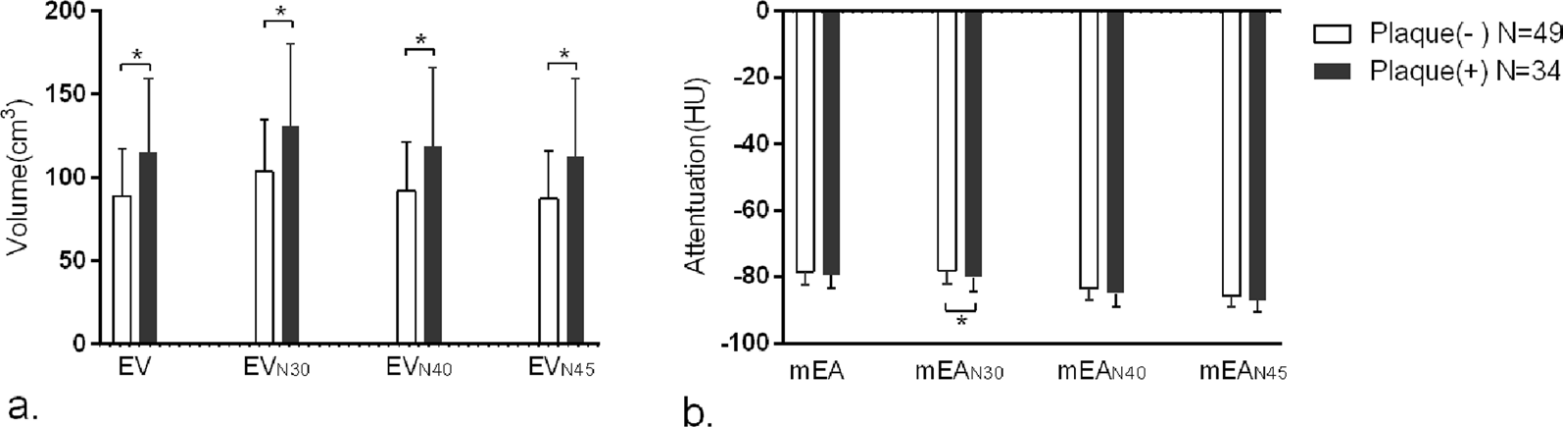
Comparison of EAT measurements between patients with and without coronary plaques. Data obtained from CCTA and RCCT both demonstrated a significantly larger EV in the plaque ( +) group than the plaque ( −) group (**a**). A significant difference in mEA was shown only on RCCT using N30 (**b**), the result of mEA measured using N40 and N45 on RCCT was same as that measured on CCTA

The results of from the EAT measurements based on the CCTA image showed that the EV in the plaque ( +) group (115.6 ± 44.1 cm^3^) was significantly larger than that of plaque ( −) group (88.9 ± 28.6 cm^3^, *P* = 0.003; Fig. 4a), and the mEA of the two groups were similar (plaque ( +) vs. ( −): − 79.5 ± 3.8HU vs. − 78.3 ± 4.2 HU, *P* = 0.204) (Table 1). When using the RCCT images and the same attenuation thresholds, the EV_N30_ of the plaque ( +) group was significantly larger than that of the plaque ( −) group (131.2 ± 49.6 cm^3^ vs. 103.7 ± 31.2 cm^3^, *P* = 0.008), but the mEA_N30_ of the plaque ( +) group was significantly lower than that of the plaque ( −) group (− 80 ± 4.4 HU vs. − 78 ± 4 HU, *P* = 0.030) (Fig. 4b), which was inconsistent with that of CCTA. After adjusting the attenuation threshold to N40 and N45, the comparison of EF measurements between the two groups was consistent with that of CCTA, whether EV or mEA. The differences in EV between the plaque ( +) and plaque ( −) groups were still significant (*P* = 0.006 and 0.009, respectively), but the differences in mEA were not significant (*P* = 0.092 and 0.075, respectively) (Fig. 4).

## Discussion

The present study validated that non-contrast RCCT could be used to quantify EV and mEA, though EV might be overestimated if the same upper threshold of − 30 HU was adopted as most previous studies used. By adjusting the upper threshold, the consistency of EV measured by RCCT with that measured by CCTA could be improved substantially, but this was not the case for mEA. Although the bias of mEA increased, the same results were obtained when comparing mEA between the two groups with and without coronary plaques. Interestingly, the mEA measured on non-contrast RCCT using N30 was sensitive enough to detect the differences in EAT characteristics between the groups with or without coronary plaques. The quantification of mEA might be more sensitive for revealing latent pathophysiological characteristics than the quantification of EV.

ECG-gated cardiac CT is considered to be the most accurate method to quantify EV because of the high resolution and true volume coverage, allowing also the assessment of mEA [18, 19]. When CT is used to measure fat, two necessary steps to measure EAT are segmentation of the pericardium and the following filter of pixels with a specific attenuation threshold of fat; the former determines the outer boundary, and the latter determines the inner boundary adjacent to the myocardium and coronary vessels [13, 20]. Previously, CCTA and CCS, both of which use ECG gating, were the most commonly used imaging techniques in this category [21]. However, it seemed that the heartbeat hindered the measurement procedure. Physiologically, the pericardium anchors the heart by attaching to the sternum, diaphragm and anterior mediastinum [22]. The inelastic characteristics ensure that the depiction of the pericardium is not affected by cardiac cycles in nongated imaging, which was verified in the segmenting step of this study (Fig. 1). With a relatively static outside boundary, motion of the inner boundary during the cardiac cycle could cause an error in EAT measurement. However, the EV assessed on diastolic and systolic CCTA reconstructions was not significantly different [23]. The EV from the systolic and diastolic phases was interchangeable when the other parameters were kept consistent. Thus, without ECG gating, RCCT could be used as an alternative method to assess EV [24–26].

Although there are abundant reports, the predefined thresholds for fat tissue are frequently inconsistent. The lower threshold is usually set at − 250 HU or − 190 HU, and the upper threshold is set at − 45 HU, − 30 HU, or − 15 HU [23, 24, 27, 28]. Therefore, it was not possible to compare the results from different studies. Among those studies, the range of (− 190 HU, − 30 HU) was the most commonly used range, and we defined the results of this range measured on CCTA as a reference. The inconsistency of the threshold was also observed in two previous studies that compared EV quantification-based non-ECG gated CT with ECG gated cardiac-CT [14, 27]. Simon-Yarza, I. et al. reported concurrent findings on the reliability between the two approaches using the threshold range of (− 195 HU, − 45 HU) [27]. Nagayama, Y et al. found that although the EV measured by nongated CT was approximately 30% higher, it demonstrated a strong correlation with gated CT using the threshold of (− 190 HU, − 30 HU) [14], which was consistent with our results. This problem hinders the longitudinal observation or retrospective analysis of EAT changes unless the same examination was conducted every time. However, both CCTA and RCCT have indications and limitations, and the available database would be appreciably expanded if the consistency of EAT measurements between them could be improved.

The present study demonstrated that adjusting the threshold could improve the consistency between EV measured by RCCT and that measured by CCTA. Bucher, A. M et al. systematically analyzed the influence of technical parameters on the quantification of EV on cardiac CT and found that threshold adjustments, especially the upper level, could make volumetry from different series comparable [23]. As shown in the CT attenuation histogram of EAT (Fig. 1c, d), the frequency near the upper threshold was approximately 20 times higher than that near the lower limit. Hence, the adjustment of the upper limit of the threshold could affect the number of pixels included and reduce the systematic bias. The latest research shows that pericoronary fat enhances approximately 4.3 HU with iodinated contrast when comparing precontrast coronary with postcontrast scanning [29]. These pixels enhanced to exceed to upper limit would be excluded when measured in contrast images. Therefore, the screened EF in CCTA which we took as reference was part of that screened in RCCT us the same threshold.

EA was reported as a measure of fat composition that might indicate the atherosclerotic process [15]. Decreased mEA and increased EV are associated with higher cardiovascular risk [30]. Recent report suggested that mEA, but not EV, is an independent predictor of obstructive CAD and high-risk plaques [31]. Quantified as the mean attenuation of screened EAT voxels, mEA is obviously affected by EV and the consistency of mEA is based on the premise of EV consistency measured by two technologies. When the volume of included EAT were more consistent between the two techniques, and the results about comparison of mEA between the two groups were unified. Remarkably, we found that the difference in mEA between patients with or without plaques was only detected using N30 in RCCT, but not for N40 and N45, indicating that the EAT ranging from − 40HU to − 30HU might be responsible for the significant difference between the two groups and also reminding us that histogram analysis of mEA would be helpful, such as percentiles of EA. Besides, the difference was neither found in CCTA, suggesting that the enhancement of EAT related to metabolic abnormalities and inflammation should considered [18]. As the most promising subsegment of EAT, pericoronary fat was found to increase its density after contrast administration [29]. Further research is required to determine whether and how EAT contrast-enhanced in physiological or pathological conditions.

The current study had limitations. First, we did not try to determine the optimal threshold or provide a recommended threshold. CT attenuation varies by equipment manufacturer, performance, and scan parameters. There are currently no endorsed guidelines to quantify EAT, even though we defined EV measured on CCTA as a reference because it was widely used in previous studies. We proposed the approach of threshold adjustment to reduce the differences in EAT measurements between the different examination protocols. Second, the number of patients was small, and the patients with coronary plaques were in early stages and asymptomatic. The predictive efficacy of EAT measurements for CAD was not explored. Third, our results may not be applicable to patients with high heart rate because metoprolol was taken for patients with heart rate > 65 beats/min before CT examination in our study.

## Conclusion

Our study demonstrates that threshold adjustment is necessary when measuring EF with non-contrast RCCT. Comparing with CCTA, the application of more negative threshold improves the consistency of EV measurements and provides a consistent result when comparing EF measurements between groups, although the bias of mEA increases. More studies are needed to reveal the subtle change of EA after contrasted enhancement and using more accurate method such as histogram analysis.

## Data Availability

The datasets used and/or analyzed during the current study are available from the corresponding author on reasonable request.

## Abbreviations

CAD: Coronary artery disease
CCS: Coronary calcium score
CCTA: Coronary CT angiography
CT: Computed tomography
ECG: Electrocardiogram
EAT: Epicardial adipose tissue
EA: EAT attenuation
mEA: Mean EAT attenuation
EV: EAT volume
HU: Hounsfield unit
LOA: Limits of agreement
RCCT: Routine chest CT
SD: Standard deviation
